# Total Temporomandibular Joint Replacement with Customized Prosthesis in Craniofacial Malformations: A Scoping Review

**DOI:** 10.3390/medicina62091717

**Published:** 2026-09-07

**Authors:** Bastian Abarzúa, Maria Ignacia Oporto, Víctor Ravelo, Erick Vargas, Florencio Monje-Gil, Sergio Olate

**Affiliations:** 1 Faculty of Dentistry, Universidad de Concepción, Concepción 4070409, Chile; bastianabarzua@gmail.com; 2 Hospital Intercultural Kallvu Llanka, Cañete 4390000, Chile; ignaciaoportoagouborde@gmail.com; 3 GIPO, Facultad Ciencias de la Salud, Universidad Autónoma de Chile, Temuco 4810100, Chile; victor.ravelo.s@gmail.com; 4 PhD Program and Center of Excellence in Morphological and Surgical Studies (CEMyQ), Universidad de La Frontera, Temuco 4811230, Chile; 5 Facultad de Odontología, Universidad San Sebastián, Concepción 4080871, Chile; erick.vargass@uss.cl; 6 Division of Oral and Maxillofacial Surgery, C.H.M Hospital, Chillán 3810525, Chile; 7 Department of Oral and Maxillofacial Surgery, Medical School Badajoz, 06006 Badajoz, Spain; fmonje@oralmaxilofacial.com; 8 Department of Oral Diagnosis, Division of Oral and Maxillofacial Surgery, Faculty of Dentistry, State University of Campinas, Piracicaba 13083-970, SP, Brazil

**Keywords:** craniofacial abnormalities, temporomandibular joint replacement, customized prostheses, orthognathic surgery

## Abstract

*Background*: Craniofacial malformations may affect maxillomandibular growth and TMJ function. Customized total TMJ replacement has been used for reconstruction in selected patients with severe congenital TMJ deficiencies. *Objectives*: To map and characterize the evidence on indications, surgical and prosthetic characteristics, associated procedures, outcomes, complications, and knowledge gaps of customized total TMJ replacement in patients with craniofacial malformations. *Methods*: This scoping review followed PRISMA-ScR guidelines. PubMed, Embase, Scopus, and Web of Science were searched from 1994 to 7 August 2026, with complementary searches in Google Scholar and OATD. Study selection and data extraction were independently performed by two reviewers, and findings were synthesized descriptively. *Results*: Seven studies comprising 36 patients aged 9–59 years were included. Forty-three TMJ replacements were performed (29 unilateral and 7 bilateral), most commonly using customized TMJ Concepts prostheses. Twenty-five patients (69.4%) had undergone previous surgery. Orthognathic surgery was part of the treatment pathway in all studies. Reported outcomes included mouth opening, facial symmetry, occlusion, and mandibular function; however, outcome reporting was heterogeneous, limiting attribution of these outcomes specifically to TMJ replacement. *Conclusions*: Evidence remains limited and heterogeneous, precluding conclusions regarding effectiveness, safety, predictability, or superiority. Prospective studies with standardized outcomes and long-term follow-up are needed.

## 1. Introduction

Craniofacial malformations are considered prevalent conditions in the pediatric population [1]. These anomalies may lead to multiple complications, including midfacial deficiency, macroglossia, micrognathia, and microsomia, affecting the function of the middle and lower facial thirds and compromising essential aspects such as proper breathing and overall quality of life [2].

Congenital deformities affecting the temporomandibular joint (TMJ) originate during the first trimester of intrauterine life and manifest as progressive alterations in the growth of the mandibular condyle and its associated articular components, resulting in hypoplasia and, less frequently, condylar aplasia [3]. Transforming growth factor beta (TGF-β) contributes to mandibular development through its role in Meckel’s cartilage [4]. In this context, hemifacial microsomia can be characterized using various classification systems that describe the extent and severity of structural developmental deficiencies [5,6]. The OMENS classification systematically evaluates five anatomical components: orbit, mandible, ear, facial nerve, and soft tissues, enabling standardized characterization of hemifacial microsomia and other craniofacial malformations [7].

The management of congenital deformities affecting the TMJ is complex and requires a comprehensive therapeutic approach aimed at achieving functional and aesthetic outcomes. Before the development of total alloplastic prostheses, TMJ reconstruction in patients with craniofacial malformations was primarily performed using autologous bone grafts, including costochondral and sternoclavicular grafts and vascularized fibular flaps [8]. Because these conditions may also involve alterations in the size, shape, and position of the maxillomandibular complex, adjunctive procedures such as orthognathic surgery may be incorporated into the treatment strategy [9].

In the early 1990s, Wolford et al. [10] introduced the concept of customized total joint prostheses, designed specifically for each patient, allowing optimal anatomical adaptation, improved stability, and enhanced functional outcomes. Subsequently, Mercuri et al. [11,12] further refined these systems by incorporating advancements in design, materials, and manufacturing techniques, including 3D printing and additive manufacturing processes to achieve precise and reproducible fitting.

Recent reviews have addressed total TMJ replacement with customized prostheses in specific craniofacial conditions or surgical settings. However, the evidence across craniofacial malformations has not been comprehensively mapped in terms of clinical indications, prosthetic characteristics, associated procedures, outcomes, complications, and knowledge gaps [9,13].

Alloplastic TMJ prostheses have been used as an alternative for the management of severe degenerative and congenital joint disorders [14]. Advances in virtual surgical planning (VSP) have also facilitated the integration of customized TMJ reconstruction with orthognathic and other reconstructive procedures in complex craniofacial cases [15].

A scoping review is therefore appropriate to systematically map this heterogeneous evidence, characterize current clinical applications and reported outcomes, and identify knowledge gaps without drawing conclusions regarding treatment effectiveness or safety. Accordingly, this scoping review aimed to map and characterize the available evidence on the clinical indications, prosthetic and surgical characteristics, associated procedures, reported outcomes, complications, and knowledge gaps related to customized total TMJ replacement in patients with craniofacial malformations.

## 2. Materials and Methods

This scoping review was conducted and reported in accordance with the Preferred Reporting Items for Systematic Reviews and Meta-Analyses Extension for Scoping Reviews (PRISMA-ScR) guidelines [16]. The review question was formulated using the Population-Concept-Context (PCC) framework as follows: What are the indications and reported clinical outcomes of total TMJ replacement using customized prostheses in patients with craniofacial malformations?

The PCC framework was defined as follows: Population, patients with craniofacial malformations; Concept, total TMJ replacement using customized prostheses; and Context, clinical application of customized total TMJ replacement in patients with craniofacial malformations, including its indications, prosthetic characteristics, associated surgical procedures, and reported clinical outcomes. No protocol for this scoping review was prospectively registered or published.

### 2.1. Eligibility Criteria

Human clinical studies with prospective or retrospective designs and case series evaluating total TMJ replacement using customized prostheses in patients with craniofacial malformations were included. Eligible conditions comprised congenital craniofacial malformations associated with mandibular hypoplasia and/or developmental abnormalities of the TMJ, including hemifacial microsomia, craniofacial microsomia, Goldenhar syndrome, Treacher Collins syndrome, Nager syndrome, craniofacial clefts, and other related congenital mandibular deficiencies.

Considering the rarity of these conditions and the exploratory nature of this scoping review, case series including three or more eligible patients with craniofacial malformations were included to identify consistent patterns in clinical indications, intervention characteristics, and reported outcomes. The three-patient threshold was applied as a pragmatic eligibility criterion rather than as an established methodological definition of a case series. Individual case reports were excluded because, although they may provide clinically relevant observations, their limited sample size does not allow the identification of reproducible clinical patterns. In studies with mixed populations, the threshold referred specifically to the number of eligible patients with craniofacial malformations whose data could be independently identified and extracted [17,18].

Although some potentially relevant individual case reports were excluded, particularly in the context of rare conditions, priority was given to ensuring a minimum level of consistency and identification of clinical patterns. This decision favored the coherence of the collected evidence over the inclusion of findings derived from individual cases.

Due to the heterogeneity of the included studies, patients with different craniofacial malformations, prosthesis types, and adjunctive procedures were analyzed together using a descriptive approach consistent with the exploratory nature of this scoping review.

Studies conducted in animals or cadavers, narrative and systematic reviews, editorials, letters to the editor, technical notes, conference abstracts, and studies without full-text availability were excluded.

### 2.2. Information Sources and Search Strategy

An electronic systematic search was conducted in PubMed, Embase, Scopus, and Web of Science from 1994, the year of publication of the first identified study on customized TMJ prostheses [10], through 7 August 2026. No language restrictions were applied. Additionally, complementary searches were conducted in Google Scholar and Open Access Theses and Dissertations (OATD) and were last updated on 7 August 2026, to identify potentially relevant records not retrieved through the bibliographic databases. Because of the large number of results retrieved by Google Scholar and its algorithmic relevance ranking, the first 200 results ranked by relevance were screened using the same eligibility criteria applied to the bibliographic databases. In OATD, all records retrieved using the adapted search strategy were screened. The reference lists of included studies and relevant reviews were manually examined, and citation tracking of the included studies was performed to identify additional potentially eligible publications.

The search terms, combined using Boolean operators AND/OR, were as follows:

(Temporomandibular joint replacement OR Total temporomandibular joint replacement OR TMJ replacement OR TMJR OR Temporomandibular joint prostheses OR Alloplastic temporomandibular joint OR Patient-specific prostheses OR Patient-fitted prostheses OR Customized prostheses OR Custom-made prostheses OR Extended TMJ prosthesis) AND (Craniofacial malformations OR Craniofacial anomalies OR Craniofacial abnormalities OR Craniofacial deformities OR Craniofacial syndromes OR Hemifacial microsomia OR Craniofacial microsomia OR Oculo-auriculo-vertebral spectrum OR Goldenhar syndrome OR Treacher Collins syndrome OR Nager syndrome OR Congenital mandibular hypoplasia).

The general strategy was adapted to the syntax, search fields, and controlled vocabulary available in each database. The complete reproducible strategies, together with the exact search dates and number of records retrieved, are provided in the Supplementary Material.

### 2.3. Study Selection and Data Extraction

All identified references were imported into Mendeley 2.90.0 (Elsevier, London, UK), where duplicate records were automatically removed. Two independent investigators subsequently screened titles and abstracts according to pre-defined eligibility criteria. Inter-rater agreement was assessed using Cohen’s kappa coefficient (κ = 0.82) after a two-week calibration process. Disagreements were resolved by discussion or consultation with a third reviewer. Potentially eligible articles were independently assessed in full text by the same reviewers, without masking author or journal information.

Data extraction was independently performed by two reviewers using a standardized form. Extracted variables included: (a) study population data (number of patients, age, sex, and type of craniofacial anomaly); (b) methodological and clinical characteristics (study design, surgical technique, type of prosthesis, adjunctive procedures, indication for total TMJ replacement, previous reconstructive procedures, and unilateral or bilateral replacement); and (c) reported clinical outcomes (follow-up, mouth opening, pain, complications, functional and aesthetic outcomes, subsequent procedures, prosthesis revisions, and explantations). No inferences were made from information that was not explicitly reported by the original authors. The authors of the included studies were not contacted for additional information; data were extracted exclusively from the information available in the publications.

### 2.4. Data Synthesis and Methodological Assessment

Data were synthesized descriptively according to the clinical and methodological characteristics of the included studies. Results were organized in evidence tables describing study populations, indications for joint replacement, reconstructive history, prosthetic characteristics, associated surgical procedures, follow-up duration, clinical outcomes, and reported complications.

Because of heterogeneity in diagnoses, patient characteristics, prosthetic systems, concomitant surgical procedures, outcome variables, and follow-up periods, no quantitative synthesis or meta-analysis was performed. Outcomes were interpreted descriptively at the study level, without indirect comparisons between prosthetic systems or surgical procedures.

In studies in which total TMJ replacement was performed together with orthognathic surgery or other reconstructive procedures, outcomes were considered results of the combined surgical treatment when the available data did not allow the independent effect attributable to joint replacement to be determined.

A formal risk-of-bias or methodological quality assessment of individual studies was not performed, considering the scoping review design and its objective of mapping and characterizing the available evidence rather than determining intervention effectiveness or safety. Accordingly, the findings were synthesized and interpreted descriptively and were not used to establish effect estimates, compare interventions, or formulate conclusions regarding efficacy, safety, predictability, or therapeutic superiority of customized total TMJ replacement. Methodological and reporting limitations of the evidence sources were explicitly considered when interpreting the findings and in the Discussion.

## 3. Results

**Figure 1:**
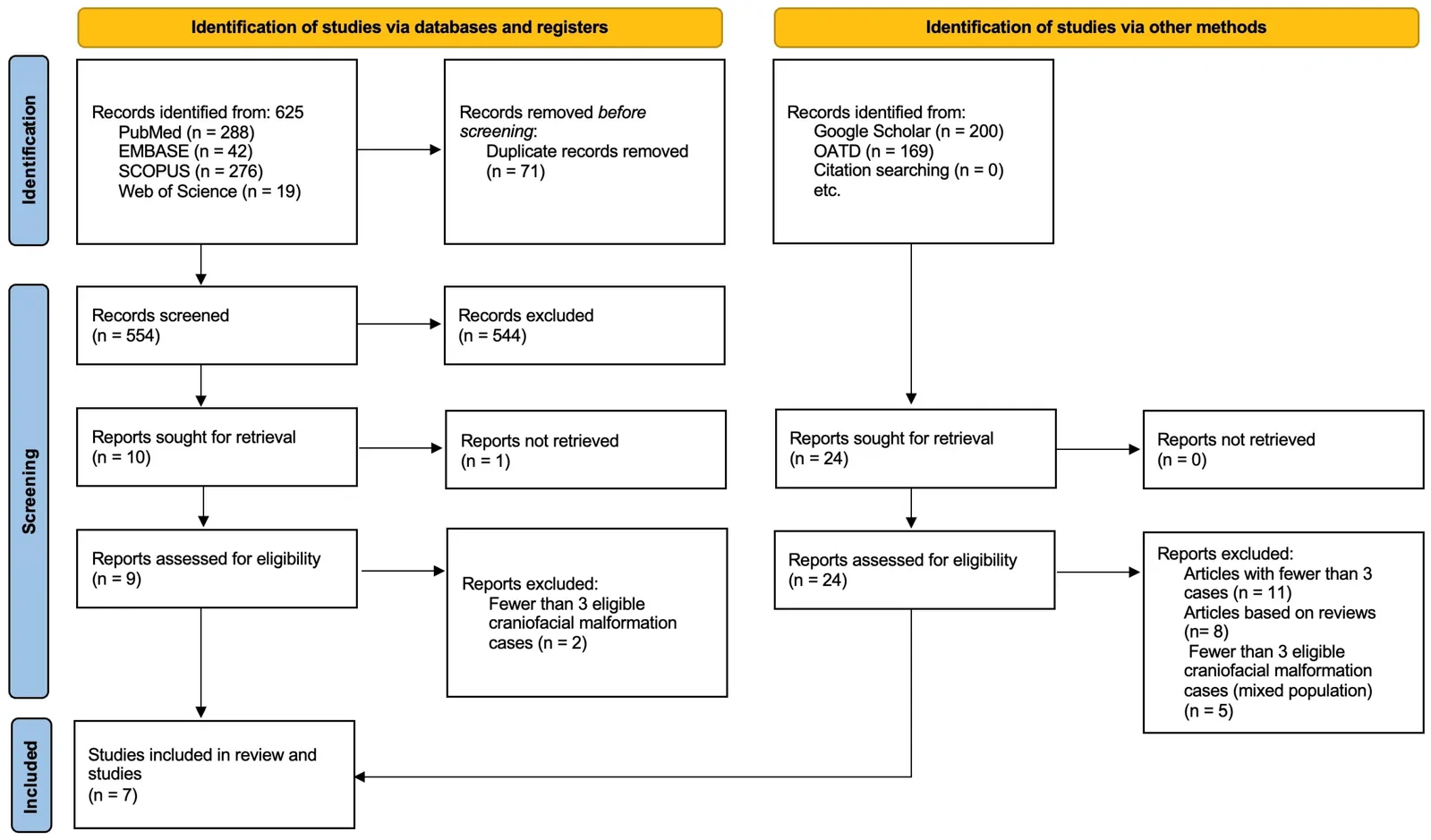
PRISMA-ScR flow diagram illustrating the study selection and screening process.

A total of 994 records were identified, including 625 from bibliographic databases and 369 from complementary sources (200 from Google Scholar and 169 from OATD). After duplicate removal and title/abstract screening, 10 reports were selected for review. One report could not be retrieved, resulting in 9 articles assessed for eligibility. Of these, two studies were excluded because they included fewer than three eligible patients with craniofacial malformations. Ultimately, 7 studies [19,20,21,22,23,24,25] met the eligibility criteria and were included in the final analysis (Figure 1). No additional eligible studies were identified through the gray literature search.

The selected studies included a total of 36 subjects. Regarding sex distribution, 15 were male and 21 were female, with an age range between 9 and 59 years. In terms of reported diagnoses, hemifacial microsomia was the most prevalent condition, followed by Goldenhar syndrome, Treacher Collins syndrome, and bilateral facial cleft associated with congenital ankylosis. The primary indications for customized TMJ replacement included severe mandibular and TMJ deficiencies, failed previous reconstructive procedures, and complex craniofacial malformations requiring definitive skeletal reconstruction. The included studies were conducted in the United States, Sweden, Greece, Finland, Spain, and Germany (Table 1).

Among the seven analyzed studies (Table 2), a total of 43 TMJ replacements were performed in 36 patients, including 29 unilateral and 7 bilateral procedures. The most frequently used prosthesis was the customized TMJ Concepts system, followed by a Zimmer Biomet prosthesis. In contrast, two studies [23,24] reported the use of customized prostheses without specifying the design or commercial brand.

Orthognathic surgery was reported in all included studies, either as a concomitant procedure or as a previous intervention before TMJ reconstruction. Bimaxillary orthognathic surgery was the most frequently reported adjunctive procedure, whereas genioplasty was described in only two studies.

Regarding pre and postoperative mouth opening, considerable heterogeneity was observed among the included studies and according to the type of craniofacial anomaly. Only three studies [20,22,24] reported both preoperative and postoperative quantitative measurements, with preoperative values ranging from 18.3 to 36.3 mm. In contrast, other studies reported only postoperative mouth opening values or did not provide complete preoperative measurements, limiting direct assessment of functional changes over time. In some cases, maximum interincisal opening could not be measured preoperatively because of severe mechanical limitations or pain.

In terms of postoperative outcomes, six of the seven included studies reported postoperative maximum interincisal opening values ranging from 30 to 40 mm, with follow-up periods between 12 and 75 months. One study [21] did not provide postoperative mouth-opening data. However, only three studies [20,22,24] reported both preoperative and postoperative measurements, whereas the remaining studies provided only postoperative values or incomplete baseline data. Consequently, the ability to assess functional changes over time was limited, and the reported postoperative mouth-opening values should be interpreted as descriptive findings rather than evidence of consistent functional improvement across all patients.

Several included studies qualitatively reported improvements in facial symmetry, occlusion, and mandibular function following customized TMJ replacement. However, standardized objective assessment methods and patient-level data were inconsistently reported, limiting quantitative comparisons across studies. Regarding procedures performed prior to joint replacement, 25 of the 36 patients (69.4%) had undergone previous surgical interventions, with costochondral grafts being the most frequently reported, followed by non-vascularized autogenous grafts. Six of the seven included studies reported joint pain associated with chronic dysfunction before TMJ replacement, whereas ankylosis was described in only one study [21].

Following TMJ replacement, two studies reported pain associated with postoperative complications. In the study by Humphries et al. [22], one patient developed external auditory canal and tympanic membrane perforation, another experienced temporal branch facial nerve neuropraxia, and a third presented permanent injury to the frontal branch of the facial nerve. In the study by Hodzic et al. [23], one prosthetic infection required removal and subsequent replacement of the TMJ prosthesis, representing the only reported case of prosthesis explantation among the included studies.

Additionally, Hodzic et al. [23] reported three patients who developed obstructive sleep apnea during postoperative follow-up and required management with continuous positive airway pressure (CPAP) (Table 3). Westermark et al. [19] described one case of TMJ condylar dislocation that did not require further surgical intervention. No additional cases of mechanical failure, implant loosening, or prosthesis displacement were reported during the follow-up period.

## 4. Discussion

Congenital joint anomalies directly impact craniofacial development and require early diagnosis and management. Mandibular hypoplasia may lead to airway reduction, feeding difficulties, speech impairments, and limitations in oral hygiene, among other complications, significantly affecting patients’ quality of life [26]. Among the most challenging deformities in cranio-maxillofacial surgery are hypoplastic asymmetries, with severe cases of hemifacial microsomia (HFM) being the most frequently encountered [21].

Advances in 3D technologies have optimized the planning and execution of complex reconstructions in maxillofacial surgery, particularly in the TMJ, where alloplastic prostheses represent a viable alternative for managing both degenerative and congenital joint disorders [19]. Traditionally, costochondral grafts have been considered the first-line option for condylar reconstruction; however, their use is limited by their non-vascularized nature, the difficulty in managing 3D defects of varying size [27], and the need for a donor site, which increases morbidity and cost. Several studies [28,29] have demonstrated that the growth of costal grafts is unpredictable, potentially resulting in insufficient or excessive growth and, in some cases, requiring additional interventions to correct deformities. Similarly, distraction osteogenesis, as an alternative therapeutic approach, presents limitations in extensive defects and depends on active patient compliance and precise vector control to achieve appropriate directional movement at the level of the mandibular condyle [8].

Alloplastic TMJ prostheses have been used in maxillofacial surgery for more than two decades; however, evidence regarding their application in patients with syndromic congenital anomalies affecting the joint remains limited. Evidence derived predominantly from adult or degenerative TMJ populations should therefore be considered indirect and should not be extrapolated to patients with congenital craniofacial malformations. Wolford et al. [9], in a systematic review, reported that the combination of alloplastic prostheses and orthognathic surgery may facilitate the correction of skeletal facial discrepancies in patients with congenital malformations. Furthermore, total joint replacement using alloplastic prostheses (condyle–fossa unit) has been associated with favorable long-term stability, reduced donor-site morbidity, and shorter operative times compared with autologous grafts. Long-term follow-up studies have also reported high levels of skeletal and occlusal stability, together with substantial reductions in pain after treatment [12,30].

Previous systematic reviews addressed more specific clinical questions. Wolford et al. [9] evaluated patient-fitted TMJ replacement combined with orthognathic surgery in patients with congenital craniofacial deformities, whereas Arif et al. [13] focused exclusively on extended TMJ replacement in hemifacial microsomia. In contrast, the present scoping review maps customized total TMJ replacement across different craniofacial malformations, characterizing clinical indications, prosthetic systems, associated procedures, reported outcomes, complications, and knowledge gaps. This broader approach was intended to characterize the available evidence rather than assess treatment efficacy or comparative effectiveness, given the heterogeneity and predominantly descriptive.

TMJ reconstruction combined with alloplastic mandibular reconstruction in severe cases of HFM has report functional outcomes in skeletally mature patients, representing a viable alternative for individuals in whom autologous reconstructions have failed [21]. Moreover, the incorporation of adjunctive procedures such as orthognathic surgery enables 3D correction of the maxilla and mandible, potentially increasing airway volume and reducing the risk of obstructive sleep apnea (OSA) [31]. In our review, orthognathic surgery was reported as part of the treatment strategy in all included studies. In Hodzic et al. [23], four of seven patients had undergone orthognathic surgery before TMJ reconstruction. Notably, three patients in that series developed OSA during postoperative follow-up and required management with continuous CPAP. This finding should be interpreted cautiously, as obstructive sleep apnea in patients with craniofacial malformations is multifactorial and cannot be attributed to surgical factors based on a single uncontrolled case series. Further studies are needed to clarify the relationship between TMJ reconstruction, orthognathic surgery, airway changes, and postoperative respiratory outcomes.

The included studies reported postoperative functional outcomes in patients with complex craniofacial malformations. However, the available evidence was derived predominantly from case series and should be interpreted cautiously. Although postoperative mouth-opening values and improvements in facial symmetry, occlusion, and mandibular function were reported in several studies, heterogeneity in outcome reporting precluded a consistent synthesis of the available evidence. Because orthognathic surgery was performed either concomitantly or as a previous procedure in all included studies, the independent contribution of customized TMJ replacement to the reported functional and aesthetic outcomes cannot be determined.

In this context, total TMJ replacement may be considered a reconstructive option for selected patients with complex craniofacial malformations. Notably, the sample included patients from 9 years of age, and 25 of 36 patients (69.4%) had undergone previous surgical interventions, including costochondral grafts, autogenous reconstructions, and other corrective procedures, before ultimately requiring TMJ replacement.

The use of TMJ prostheses in skeletally immature patients remains controversial. Liu et al. [32] provided a comprehensive review outlining the transition from autogenous reconstruction to alloplastic replacement, highlighting the potential of current systems in managing complex cases. Similarly, Mercuri et al. [33] emphasized the advantages of alloplastic systems compared to costochondral reconstruction. Although Sinn et al. [34] reported favorable outcomes and stability in a pediatric clinical series, the available evidence remains insufficient to establish recommendations regarding the routine use of TMJ prostheses in skeletally immature patients. Therefore, treatment should be individualized, considering residual craniofacial growth and the limited evidence on long-term outcomes.

In 2002, Mommaerts and Nagy [35] highlighted the limited scientific evidence supporting early distraction osteogenesis (DO) as a superior approach in terms of outcomes and morbidity when compared with alternative reconstructive strategies. Subsequently, Meazzini et al. [36] reported that patients with HFM treated with DO demonstrate an initial improvement in facial symmetry; however, recurrent asymmetry was observed during a 5-year follow-up period. Similar findings were reported in a later study comparing patients with HFM treated with and without DO [37].

The available evidence describes the combined use of alloplastic TMJ reconstruction and orthognathic surgery in selected patients approaching or after skeletal maturity; however, the limited and heterogeneous evidence does not allow conclusions regarding optimal treatment timing, surgical sequencing, or comparative effectiveness. TMJ malformations are often associated with deficiencies in the development of the external ear, as well as surrounding muscular and cutaneous tissues, resulting in a depression of the affected facial region and contributing to asymmetry [4,38]. Soft tissue conditions remain poorly described in the literature, despite their clinical relevance. The absence of adequate soft tissue in the joint region may influence prosthetic design, particularly when reconstruction of the superior segment (zygomatic component) is required for placement of the glenoid fossa component, or when restoration of lower facial contour and symmetry necessitates increased volume in the ramus-condyle unit. Prosthesis placement requires adequate soft tissue coverage and closure planes, which are often difficult to achieve in complex anomalies and may predispose to complications. One recommendation in prosthetic design is to opt for normal or reduced dimensions to facilitate proper soft tissue closure and avoid excessive volume, as inadequate closure may increase the risk of transcutaneous exposure of the condylar component [23,39]. Additionally, the use of abdominal fat grafting may be considered in such cases; however, risks related to donor site morbidity and wound healing complications must also be considered [40].

However, these outcomes were reported inconsistently and were predominantly based on qualitative assessments. Similar observations have been reported by Wolford et al. [30] and Mercuri and Giobbie-Hurder [12], who described high levels of skeletal stability and pain control following total alloplastic TMJ replacement. Although relatively few major complications were reported, the included studies described events such as facial nerve injury, prosthetic infection requiring explantation, obstructive sleep apnea, and displacement of prosthetic components. Consequently, the available evidence suggests that TMJ replacement may represent a reconstructive option for selected patients with complex craniofacial malformations; however, the limited number of studies and the predominance of case series warrant cautious interpretation of these findings.

The mandible-first and maxilla-first surgical sequences have both been described in association with orthognathic surgery and TMJ prosthetic reconstruction. However, the available evidence does not allow conclusions regarding the superiority or predictability of a specific surgical sequence [41,42]. A meta-analysis by Zhao et al. [43] reported no significant differences in surgical accuracy in either linear or rotational terms between these approaches, suggesting that the decision should be individualized based on surgical planning and clinical conditions, particularly in cases involving TMJ reconstruction. In contrast, surgical protocols involving initial prosthetic placement followed by maxillary osteotomy have been described in complex cases involving severe skeletal discrepancies or TMJ prosthetic reconstruction [27,44]. However, the available evidence does not allow conclusions regarding the superiority or predictability of a specific surgical sequence.

Polley et al. [21] and Wolford et al. [9] reported functional and reconstructive improvements following combined TMJ replacement and orthognathic surgery in patients with severe craniofacial asymmetries. However, the available evidence is insufficient to determine the independent contribution of TMJ replacement or to establish recommendations regarding this combined treatment approach. Based on the available evidence, combined TMJ prosthetic reconstruction and orthognathic surgery has been reported in selected patients who have reached skeletal maturity; however, the available evidence is derived primarily from case series and is insufficient to establish definitive recommendations regarding treatment indications, protocols, or effectiveness. In this context, the limitations reported for autogenous reconstruction and distraction osteogenesis support further investigation of alternative reconstructive approaches for the management of complex craniofacial conditions.

Prosthesis longevity remains a major consideration, particularly in patients who require reconstruction before or around skeletal maturity. Previous long-term studies have reported favorable functional outcomes and implant survival [45], while Lima et al. [46] reported a pooled prosthesis survival rate of 97% (CI: 95–99%), with most failures occurring within the first 6 postoperative months. Similarly, Wood-Kurland et al. [47] described lower complication rates and improved reliability in contemporary TMJR systems. However, these findings are derived predominantly from broader adult TMJ populations and should be considered indirect evidence for patients with congenital craniofacial malformations. Long-term prosthesis survival in this specific population remains insufficiently characterized.

Therefore, limited evidence regarding very long-term outcomes in patients with high functional demands continues to restrict definitive conclusions on prosthesis longevity. Long-term survival data derived from broader TMJ replacement populations should be considered indirect evidence and cannot be directly extrapolated to patients with congenital craniofacial malformations.

The findings should be interpreted with caution given the limited and low-level evidence available. This reflects the rarity of craniofacial malformations requiring temporomandibular joint prosthetic reconstruction, with the included evidence derived predominantly from small retrospective case series without control groups. Consequently, sample sizes are small, and methodological strength is limited, restricting the robustness and generalizability of the findings. In addition, there is marked clinical heterogeneity across diagnoses (e.g., hemifacial microsomia, Goldenhar syndrome, Treacher Collins syndrome, and Nager syndrome), as well as variability in prior reconstructive history, prosthetic systems, adjunctive procedures, and follow-up duration, which prevents meaningful data synthesis. Outcome reporting was heterogeneous, with limited use of standardized functional, aesthetic, and patient reported measures, frequent absence of preoperative functional data, and potential selective outcome reporting. Follow-up periods were also variable, and publication bias toward successful outcomes cannot be excluded.

The mapped evidence highlights several knowledge gaps, including the absence of prospective comparative studies, standardized outcome definitions and measurement instruments, patient-reported outcome measures, and long-term follow-up. Future research should prioritize multicenter prospective studies with standardized outcome assessment and reporting to better characterize the indications, outcomes, complications, and long-term performance of customized TMJ replacement in patients with craniofacial malformations.

## 5. Conclusions

Available evidence is limited and derived predominantly from small retrospective case series without control groups. Customized TMJ replacement has been reported as a reconstructive approach in selected patients with craniofacial malformations involving the TMJ. However, the heterogeneity and methodological limitations of the available evidence preclude definitive conclusions regarding treatment effectiveness, safety, predictability, or superiority. Because orthognathic surgery was frequently incorporated into the treatment pathway, the independent contribution of customized TMJ replacement to the reported outcomes cannot be determined. Further prospective studies with larger samples, standardized outcome measures, and long-term follow-up are needed to better characterize treatment indications, outcomes, complications, and long-term prosthetic performance.

## Figures and Tables

**Table 1:** Description of the demographic and diagnostic characteristics of the studies included in the review.

Authors	Subjects	M/F	Age (Years)	Country	Diagnosis	Indication for Customized TJR
Westermark et al. [19]	3	0/3	18–59	Sweden	1 Goldenhar syndrome; 2 hemifacial microsomia	Major TMJ and mandibular defects unsuitable for autogenous reconstruction
Wolford et al. [20]	6	2/4	14–36	United States	Hemifacial microsomia	Severe condylar–ramus deficiency
Polley et al. [21]	10	6/4	20.0 ± 1.4	United States and Greece	Hemifacial microsomia	Failed autogenous reconstruction
Humphries et al. [22]	4	1/3	16–30	United States	2 Goldenhar syndrome; 1 hemifacial microsomia; 1 bilateral facial cleft with congenital ankylosis	Congenital TMJ deformities with mandibular deficiency
Hodzic et al. [23]	7	3/4	16–24	Finland	4 Goldenhar syndrome; 1 Treacher Collins syndrome; 1 Nager syndrome; 1 hemifacial microsomia	TMJ deformity with mandibular dysfunction
Zimmerer et al. [24]	3	1/2	9–22	Germany	2 Goldenhar syndrome; 1 non-syndromic bilateral congenital mandibular hypoplasia	Persistent mandibular deficiency after previous reconstruction
Valls-Ontañon et al. [25]	3	2/1	17–19	Spain	Hemifacial microsomia, Pruzansky–Kaban type III	Severe mandibular hypoplasia

**Table 2:** Distribution of customized prostheses, adjunctive surgical procedures, and functional outcomes of mandibular opening.

Authors	Prosthesis Used	Prosthesis Distribution	Adjunctive Surgery	Preoperative Mouth Opening (mm)	Postoperative Mouth Opening (mm)	Follow-Up (Months)
Westermark et al. [19]	Customized TMJ Concepts	2 unilateral, 1 bilateral	Bimaxillary orthognathic surgery	NR	30–38	72
Wolford et al. [20]	Customized TMJ Concepts	6 unilateral	Bimaxillary orthognathic surgery and genioplasty	36.3	39.2	75
Polley et al. [21]	Customized TMJ Concepts	9 unilateral, 1 bilateral	Bimaxillary orthognathic surgery	NR	NR	6–50
Humphries et al. [22]	Customized TMJ Concepts	3 unilateral, 1 bilateral	Bimaxillary orthognathic surgery and genioplasty	30	40	24.5
Hodzic et al. [23]	Customized prosthesis	4 unilateral, 3 bilateral	Orthognathic surgery	NR	34	NR
Zimmerer et al. [24]	Customized Zimmer Biomet	2 unilateral, 1 bilateral	Orthognathic surgery	18.3	31.67	42
Valls-Ontañon et al. [25]	Customized prosthesis	3 unilateral	Bimaxillary orthognathic surgery	NR	38.8	12

Note: NR: not reported.

**Table 3:** Description of surgical history, pain management, and postoperative adverse events in the study sample.

Authors	Previous Interventions	Previous Condylar Reconstruction	Preoperative Pain	Postoperative Pain	Postoperative Complications	Postoperative Procedures
Westermark et al. [19]	1	Costochondral graft	Joint pain and dysfunction	Absence of pain in all subjects	1. Condylar dislocation in one case	NR
Wolford et al. [20]	0	NR	Pain present in all subjects	Absence of pain in all subjects	1. Reduced final mouth opening in 2 patients	NR
Polley et al. [21]	10	Costochondral grafts	Joint pain and dysfunction	Absence of pain in all subjects	None	NR
Humphries et al. [22]	3	Non-vascularized autogenous condylar grafts	Pain present and one case of severe ankylosis	Pain present in three subjects	1. External auditory canal and tympanic membrane perforation; 2. Temporal facial nerve neuropraxia; 3. Permanent damage to the frontal branch of the facial nerve	Tympanoplasty; brow lift in patient with permanent facial nerve injury
Hodzic et al. [23]	6	Non-vascularized autogenous condylar grafts	Pain associated with chronic dysfunction	Pain present in one subject	1. Facial paresis in 4 cases; 2. Prosthetic infection in 1 case; 3. Onset of obstructive sleep apnea in 3 cases	Removal of infected prosthesis and secondary reconstruction; CPAP management for OSA
Zimmerer et al. [24]	3	Fibula free flap; mandibular distraction; costochondral graft; interpositional gap arthroplasty	Pain present in all subjects	Absence of pain in all subjects	None	NR
Valls-Ontañon et al. [25]	2	NR	NR	Absence of pain in all subjects	1. Mild, transient facial paresis	NR

Note: NR: not reported.

## Data Availability

The original contributions presented in this study are included in the article/Supplementary Material. Further inquiries can be directed to the corresponding author.

## Supplementary Materials

The following supporting information for Search Strategies can be downloaded at: https://www.mdpi.com/article/10.3390/medicina62091717/s1, Table S1. The general search strategy was adapted to the syntax, search fields, and controlled vocabulary available in each database and gray literature source. The searches covered the period from 1994 through 7 August 2026.
