# Supplementary material for: Total Temporomandibular Joint Replacement with Customized Prosthesis in Craniofacial Malformations: A Scoping Review
Source: Medicina (Kaunas). 2026 Sep 7;62(9):1717. doi: 10.3390/medicina62091717 (PMC13609089; doi:10.3390/medicina62091717)
Supplement: Supplementary file 1 [file medicina-62-01717-s001.zip › medicina-4475210-supplementary.pdf]

Supplementary Material

Table S1. The general search strategy was adapted to the syntax, search fields, and controlled vocabulary available in each database and gray literature source. The searches covered the period from 1994 through 7 August 2026.

Source	Search strategy	Records
**PubMed**	((((((((Temporomandibular joint replacement) OR (Total temporomandibular joint replacement)) OR (TMJ replacement)) OR (Temporomandibular joint prostheses)) OR (Alloplastic temporomandibular joint)) OR (Patient-specific prostheses OR Patient-fitted prostheses)) OR (Customized prostheses)) OR (Custom-made prostheses)) AND ((((((((((Craniofacial malformations) OR (Craniofacial anomalies)) OR (craniofacial abnormalities[MeSH Terms])) OR (Craniofacial deformities)) OR (Craniofacial syndromes)) OR (hemifacial microsomia[MeSH Terms])) OR (Craniofacial microsomia)) OR (goldenhar syndrome[MeSH Terms])) OR (Treacher Collins syndrome)) OR (Nager syndrome)) OR (Congenital mandibular hypoplasia))	288
**Embase**	('temporomandibular joint replacement'/exp OR 'temporomandibular joint replacement' OR 'total temporomandibular joint replacement' OR 'tmj replacement' OR 'temporomandibular joint prostheses'/exp OR 'temporomandibular joint prostheses' OR 'alloplastic temporomandibular joint' OR 'patient-specific prostheses' OR 'patient-fitted prostheses' OR 'customized prostheses' OR 'custom-made prostheses') AND ('craniofacial malformations' OR 'craniofacial anomalies' OR 'craniofacial abnormalities' OR 'craniofacial deformities' OR 'craniofacial syndromes' OR 'hemifacial microsomia' OR 'craniofacial microsomia' OR 'goldenhar syndrome' OR 'treacher collins syndrome' OR 'nager syndrome' OR 'congenital mandibular hypoplasia') AND [1994-2026]/py	42
**Scopus**	(ALL ("temporomandibular joint replacement" OR "total temporomandibular joint replacement" OR "TMJ replacement" OR "temporomandibular joint prostheses" OR "alloplastic temporomandibular joint" OR "patient-specific prostheses" OR "patient-fitted prostheses" OR "customized prostheses" OR "custom-made prostheses") AND ALL ("craniofacial malformations" OR "craniofacial anomalies" OR "craniofacial abnormalities" OR "craniofacial deformities" OR "craniofacial syndromes" OR "hemifacial microsomia" OR "craniofacial microsomia" OR "Goldenhar syndrome" OR "Treacher Collins syndrome" OR "Nager syndrome" OR "congenital mandibular hypoplasia")) AND PUBYEAR > 1994	276
**Web of Science**	("temporomandibular joint replacement" OR "total temporomandibular joint replacement" OR "TMJ replacement" OR "temporomandibular joint prostheses" OR "alloplastic temporomandibular joint" OR "patient-specific prostheses" OR "patient-fitted prostheses" OR "customized prostheses" OR "custom-made prostheses") AND ALL ("craniofacial malformations" OR "craniofacial anomalies" OR "craniofacial abnormalities" OR "craniofacial deformities" OR "craniofacial syndromes" OR "hemifacial microsomia" OR "craniofacial microsomia" OR "Goldenhar syndrome" OR "Treacher Collins syndrome" OR "Nager syndrome" OR "congenital mandibular hypoplasia")	19
**Google Scholar**	("temporomandibular joint replacement" OR "TMJ replacement" OR "temporomandibular joint prostheses" OR "alloplastic temporomandibular joint") ("craniofacial malformations" OR "craniofacial anomalies" OR "craniofacial microsomia" OR "hemifacial microsomia" OR "Goldenhar syndrome" OR "Treacher Collins syndrome" OR "Nager syndrome")	200*
**OATD**	"temporomandibular joint replacement" "TMJ replacement" "temporomandibular joint prostheses" "craniofacial malformations" "craniofacial microsomia" "hemifacial microsomia" "Goldenhar syndrome" "Treacher Collins syndrome"	169

Notes: *For Google Scholar, because of the large number of results, the first 200 results ranked by relevance were screened.; OATD: Open Access Theses and Dissertations.
